# Illinois State Dental Society

**Published:** 1886-03

**Authors:** 


### PROCEEDINGS.

### Illinois State Dental Society.

CONCLUDED FROM PAGE 611, NO. 12, VOL. XXXIX.

Dr. C. W. Spalding, St. Louis, read a paper on Inflammation in which he stated that the microscope had revealed certain conditions of the blood and of the blood vessels which are generally recognized as inflammatory; that these conditions consist of a slight enlargement of all the blood vessels involved, an increased movement of the blood column, followed by a movement slower than normal, and of the escape of both white and red blood corpuscles through the walls of the blood vessels. He said the latter process takes place by diapedesis and not by osmosis as is the case with plasma in a state of health. Also that inflammation of repair and that of destruction or disorganization, although opposite in the results produced, differ only in degree, so far as has yet been observed. In the process of repair he spoke of cicatricial tissue as being built up by the metamorphosis of white blood corpuscles, but when the escaped corpuscles were in excess of the wants of the process of repair, they accumulated, and these accumulations became the probable source of the so-called pus-corpuscles.

Dr. Black. The formation of cicatricial tissue may be observed under the microscope, and the process may thus become subject to examination by actual vision. The formation of tissue and the formation of pus are alike, except that cells favorably placed are incorporated into tissue, while those unfavorably placed go to form pus.

Inflammation means the death of the affected tissue, tissue injury being all there is of inflammation. There may be an excessive growth of tissue, or there may be a great waste of tissue elements and a corresponding accumulation of pus. Hyperemia precedes inflammation, and this condition may, or may not be followed by an inflammatory state. In hyperemia there is an increase of blood, and hyperemia may become destructive, without the happening of inflammation at all. Inflammation may also occur in the absence of any or all the usual symptoms which indicate that condition.

In hyperemia the red blood-corpuscles have passed the vessel walls, while in inflammation the white blood corpuscles are the blood elements that have escaped. This constitutes the distinguishing feature between the two conditions. They are both pathological, for in the physiological state the blood corpuscles do not escape.

Dr. Brophy thought the escape of leucocytes not necessary to an inflammatory condition, and that the hyperemic state may be a physiological one.

Dr. Ingersoll, Keokuk. Authors differ in their descriptions of what they call inflammation. Stricker says active hyperemia and cell metamorphosis constitute inflammation.

Dr. Black says hyperemia is not inflammation.

Stricker says pus-corpuscles are derived from retrograde metamorphosis of connective tissue cells, and not from blood corpuscles. Inflammation comprises a series of processes, one following another, the first of which is irritative, which Stricker calls active hyperemia. Other conditions follow, one of which is that in which the formation of pus takes place, not as a sequence of inflammation, as stated by the essayist, but as one of its proper processes. Stricker speaks of pus formation as part and parcel of the inflammatory process. By metamorphosis, we understand the conversion of one kind of tissue into another ; the destruction of tissue cell cannot properly be called a metamorphosis, retrograde or otherwise.

Dr. Black. We have hyperemia that precedes inflammation, and we have hyperemia without inflammation. Pus is formed from the metamorphosis of tissue, and not from blood corpuscles.

Previous to adjournment the officers of the Society for the ensuing year were elected and the usual closing resolutions passed.

The list of the officers elect is as follows :

President, Dr. Thos. L. Gilmer, of Quincy.

Vice-President, Dr. W. B. Woodward, of Peoria.

Secretary, Dr. J. W. Wassall, of Chicago.

Assistant Secretary, Dr. P. J. Kester, of Chicago.

Treasurer, Dr. C. B. Rohland, of Alton.

Librarian, Dr. W. B. Ames, of Chicago.

